# The Mechanisms of Sijunzi Decoction in the Treatment of Chronic Gastritis Revealed by Network Pharmacology

**DOI:** 10.1155/2020/8850259

**Published:** 2020-10-23

**Authors:** Ting Wang, Yuyin Feng, Hesong Wang, Guiyang Huo, Yanan Cai, Li Wang, Kai Yuan, Guangrui Huang

**Affiliations:** ^1^School of Life Sciences, Beijing University of Chinese Medicine, Beijing, China; ^2^Beijing Anyuanquanlv Medical Institute, Beijing, China

## Abstract

Chronic gastritis is characterized by inflammation in the gastric mucosa with a vicious circle in inflammatory cells and inflammatory mediators. Stomach adenocarcinoma would occur in the metaplastic gastric mucosa of chronic gastritis. Sijunzi decoction is a famous classical formula for the treatment of chronic gastritis. Although previous studies revealed some functions of Sijunzi decoction in treating chronic gastritis, the underlying mechanisms have not been illustrated clearly. In this study, we used network pharmacology to investigate the mechanism of Sijunzi decoction in treating chronic gastritis. Firstly, online datasets TCMSP, SWISS, and DisGeNET were used to investigate the functional mechanism of Sijunzi decoction against chronic gastritis and 18 genes were identified as targets of Sijunzi decoction in chronic gastritis. These 18 genes can be categorized into immunologically related genes and cancer-related genes. GO analysis showed that the 18 target genes were mainly enriched in angiogenesis, nitric oxide biosynthetic process, ERK1 and ERK2 cascade, cellular response to drug, and MAPK cascade. So, Sijunzi decoction alleviated chronic gastritis by inhibiting the local inflammatory response. Furthermore, we also investigated the impact of Sijunzi decoction on the peripheral blood leukocytes with our own RNA sequencing (RNA-seq) data of 11 chronic superficial gastritis patients. 102 differentially expressed genes (DEGs) were identified by comparing RNA-seq data of chronic superficial gastritis patients with healthy control groups. After performing a functional analysis on 102 DEGs and Sijunzi decoction potential targets and taking the intersection of these pathways, we found that platelet activation, angiogenesis, and pathways in cancer were candidate target pathways regulated by Sijunzi decoction. Thus, Sijunzi decoction also alleviates chronic gastritis by suppressing inflammatory response of peripheral blood leukocytes. Our results showed that Sijunzi decoction can ameliorate the local gastric inflammation and inflammations in peripheral blood leukocytes and might also reduce the incidence of stomach cancer in chronic gastritis.

## 1. Introduction

Gastritis is the inflammation of the gastric mucosa. The symptoms of gastritis include abdominal pain, vomiting, nausea, heartburn, and loss of appetite. Gastritis might lead to some kinds of complications such as stomach ulcers, stomach bleeding, and cancer [1]. The classifications of gastritis include acute gastritis and chronic gastritis depending on the duration of disease. Although the prevalence of chronic gastritis has been decreased in the past several decades especially in developed countries, it is one of the common diseases worldwide [2]. *Helicobacter pylori* infection is the most important pathogenic factor of chronic gastritis [3]. In the clinical practice, digestive endoscope is used to visualize and diagnose chronic gastritis. Histopathological examination of chronic gastritis indicates that the basic lesions are inflammatory lesions and atrophic. Thus, chronic gastritis could be divided into chronic superficial gastritis and chronic atrophic gastritis [4]. The features of chronic superficial gastritis under endoscope are bleeding mucosal spots, mucosal roughness, and mucosal erythema [5]. The features of chronic atrophic gastritis are the loss of gastric mucosal glands. The treatments of chronic gastritis are *H. pylori* eradication, proton pump inhibitors, antacids, and surgery. The purposes of the treatment are etiology eradication, symptoms palliation, and inflammatory response alleviation. However, the previous methods have adverse effects in the treatment of chronic gastritis [6]. So, it is important to discover novel methods to deal with chronic gastritis.

Sijunzi decoction is a famous classical formula in traditional Chinese medicine [7]. Four Chinese herbs were composed in Sijunzi decoction including *Radix Codonopsis pilosulae* (renshen, ginseng), *Poria* (fuling, Indian buead tuckahoe), *Rhizoma atractylodis macrocephalae* (baizhu, largehead atractylodes Rh), and *Radix glycyrrhizae* (gancao, licorice). It was used to treat various digestive diseases such as gastritis, duodenitis, and inflammatory bowel disease [8]. As for chronic gastritis, researchers have conducted some clinical trials to investigate the effects of Sijunzi decoction [9]. Tian et al. found that modified Sijunzi decoction could alleviate the symptoms of chronic atrophic gastritis such as fatigue and tiredness [10]. Gan et al. enrolled 6 randomized controlled trials to conduct meta-analysis and demonstrated that Sijunzi decoction was beneficial to chronic atrophic gastritis patients [9]. However, they have not revealed the mechanism of Sijunzi decoction in the treatment of chronic gastritis.

Network pharmacology is a new and promising subject to discover the mechanism of drugs based on network science. The network of drug and targets is very important to understand the mechanism of drugs. Combination of drugs is applied in network pharmacology because multiple pharmaceutical ingredients aim at multiple targets in the entire disease module. Chinese herbs are discovered with network pharmacology because they possess multiple components and targets. In addition, the reasons of complex diseases such as chronic gastritis are dysfunction of network in the body. Multiple molecular targets are involved in the development of chronic gastritis. So, the methodology of network pharmacology could be applied to unveil the mechanism of Chinese herbs in the treatment of chronic gastritis.

In this study, we tried to explore the mechanism of Sijunzi decoction in the treatment of chronic gastritis. Firstly, we identified the potential targets of Sijunzi decoction and chronic gastritis. Secondly, we merged the two maps to obtain the molecular targets of Sijunzi decoction in the treatment of chronic gastritis. Then, we performed functional enrichment analyses about the overlap gene to discover the biological processes. In addition, we analyzed the differentially expressed genes (DEGs) from the blood leukocytes of chronic superficial gastritis patients to validate the previous results and investigate functional mechanism of Sijunzi decoction. This study could provide novel molecular targets of Sijunzi decoction in the treatment of chronic gastritis.

## 2. Method

### 2.1. Screening of Potential Targets for Sijunzi Decoction and Chronic Gastritis

TCMSP and SWISS databases were used to identify the potential targets for Sijunzi decoction. DisGeNET dataset was used to discover the potential targets of chronic gastritis. TCMSP database (http://lsp.nwu.edu.cn/tcmsp.php) is a powerful dataset focused on traditional Chinese medicine to investigate the relationship between targets, drugs, and diseases. TCMSP database included 499 Chinese herbs, 3,311 targets, and 837 diseases. The properties of absorption, distribution, metabolism, and excretion (ADME) such as drug likeness, oral bioavailability, and half-life were also provided by TCMSP. Swiss Target Prediction database is a powerful dataset to predict the ligand bioactive molecules. DisGeNET database is a platform including genes and diseases. It could be used to investigate the molecular underpinnings of diseases, gene properties, drug adverse effects, etc.

### 2.2. Subjects and Samples

In this study, 11 patients with chronic superficial gastritis aged between 18 and 65 years old were enrolled. Gastroscopy and pathological examination were used to diagnose chronic superficial gastritis. 5 healthy volunteers aged between 18 and 65 years old were included as healthy control group. The study was approved by the Institutional Review Board of Beijing University of Chinese Medicine (JDF-IRB-2016031002).

### 2.3. Blood Leukocytes Collection and RNA Sequencing Analysis

The peripheral blood was collected from patients before treatment. 5 mL peripheral blood sample was collected in additive-free blood collection tubes. The blood leukocytes were isolated according to the protocol of the lymphocyte separation reagent (Solarbio, Beijing, China). The total RNA was extracted with TRIzol reagent (Invitrogen Life Technologies, Carlsbad, CA, USA) and stored at −80°C. The mRNA sequencing libraries were constructed with the total RNA of blood leukocytes. Illumina HiSeq 2000 platform was used to analyze the sequencing of mRNA. Then, data homogenization and quality control were performed and the transcript expression levels were evaluated with fragments per kilobase million (FPKM). The repeated sequences were removed by the RepeatMasker web server. The analysis of principal component analysis (PCA) also indicated clear distinction between chronic superficial gastritis patients and healthy control group. Differentially expressed mRNAs were explored between chronic superficial gastritis patients and healthy control group. Fold change >2 and *p* < 0.05 were significant.

### 2.4. PPI Network Construction

We conducted the protein–protein interaction (PPI) network analysis with STRING database (https://string-db.org/). STRING database is a powerful database to predict the interaction between proteins. Nodes and edges numbers and value of PPI enrichment could be assessed in STRING database. Furthermore, top hub nodes and connectivity degree of proteins could also be evaluated with PPI network in STRING database.

### 2.5. GO Analysis

Gene Ontology (GO) analysis was conducted to discover the functional characteristics of overlap DGEs in our study. GO analysis (http://www.bioinformatics.com.cn) includes biological process (BP), cellular component (CC), and molecular function (MF). BP describes the physiological or cellular functions of the selected genes. CC represents the location of gene products to perform their functions in the cell. MF means the molecular activities of the selected genes. Hypergeometric distribution test was conducted to reveal the DEGs significance in each GO entry. So, GO analysis could provide hierarchically selected gene products or genes into organized terms under graph structures.

### 2.6. KEGG Signaling Pathway Analysis

Kyoto Encyclopedia of Genes and Genomes (KEGG) pathway analysis was performed to explore the correlated functional pathways of selected genes. KEGG (http://www.kegg.jp/) is a useful bioinformatics database dealing with diseases, drugs, genes, and biological pathways. KEGG pathway map represents the experimental knowledge of cell and organism functions. In the pathway, a network containing molecular reactions and interactions is correlated with genes or gene products. At last, bubble chart was visualized on the platform.

## 3. Results

### 3.1. The Potential Targets of Sijunzi Decoction and Chronic Gastritis

**Figure 1:**
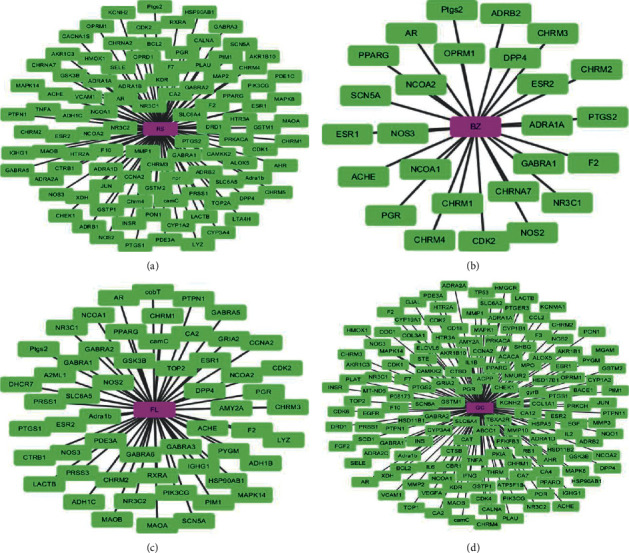
Potential targets of Sijunzi decoction. (a) *Radix Codonopsis pilosulae.* (b) *Poria.* (c) *Rhizoma atractylodis macrocephalae.* (d) *Radix glycyrrhizae*.

Sijunzi decoction was combined with 4 Chinese herbs including *Radix Codonopsis pilosulae*, *Poria*, *Rhizoma atractylodis macrocephalae*, and *Radix glycyrrhizae.* There were 100 potential targets in *Radix Codonopsis pilosulae.* As for *Poria*, there were 56 potential targets in the specific Chinese herb. In addition, *Rhizoma atractylodis macrocephalae* had 26 potential targets. At last, *Radix glycyrrhizae* had 151 potential targets. Furthermore, we combined the potential targets of 4 Chinese herbs in Sijunzi decoction and identified 177 potential targets in Sijunzi decoction (Figure 1).

**Figure 2:**
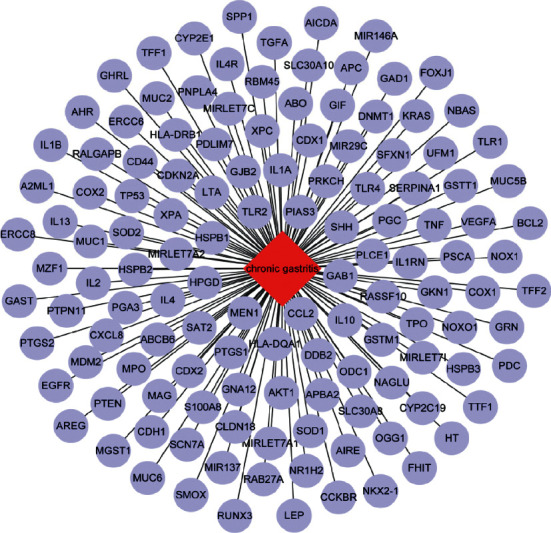
Potential targets for chronic gastritis.

In addition, we used DisGeNET dataset to discover the potential targets of chronic gastritis. Chronic gastritis possessed 129 potential targets in the dataset including EGFR, TP53, COX2, TNF, IL-1B, IL2, CXCL8, CCL2, and IL13 (Figure 2).

### 3.2. Functional Analysis of Potential Treatment Targets of Sijunzi Decoction on Chronic Gastritis

After analyzing the potential targets of Sijunzi decoction and chronic gastritis, we found 18 targets were overlapped, which can be mainly categorized into immunologically related genes and cancer-related genes (Table 1).

**Figure 3:**
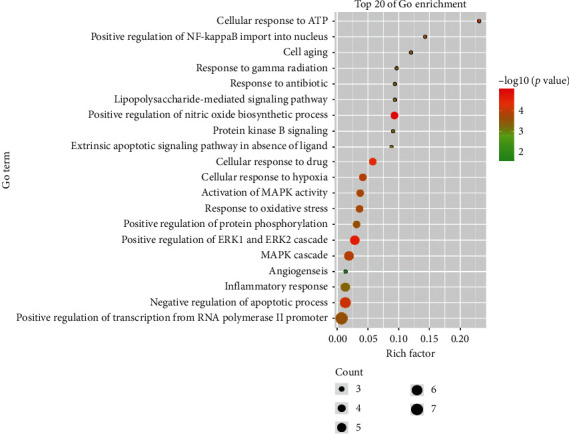
GO analysis of 18 genes.

Then, we conducted a GO analysis of these 18 targets and found that the most related biological processes were positive regulation of nitric oxide biosynthetic process, positive regulation of ERK1 and ERK2 cascade, cellular response to drug, and negative regulation of apoptotic process and MAPK cascade (Figure 3).

### 3.3. DEGs in Peripheral Blood Leukocytes of Chronic Gastritis Patients

The RNA-seq of peripheral blood leukocytes from 11 chronic superficial gastritis patients and 5 normal people was performed to investigate the inflammation in peripheral blood. 102 differentially expressed genes (DEGs) were identified by comparing gene expression level between chronic superficial gastritis patients and healthy control groups, among which 56 genes were upregulated and 46 genes were downregulated in chronic gastritis patients (Table 2).

### 3.4. Functional Analysis of Sijunzi Decoction Potential Targets and DEGs of Chronic Gastritis Patients Peripheral Bood Lukocytes

**Figure 4:**
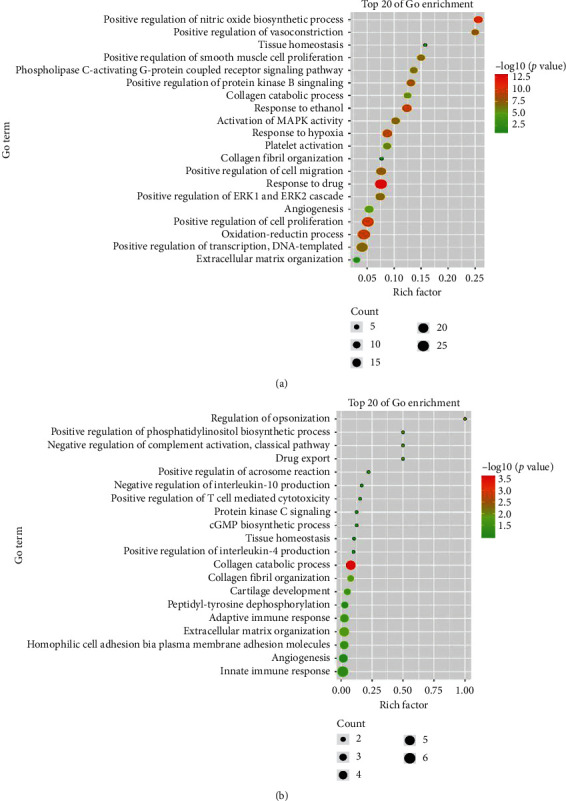
(a) GO analysis of Sijunzi decoction potential targets. (b) GO analysis of DEGs of chronic gastritis patients peripheral blood leukocytes.

**Figure 5:**
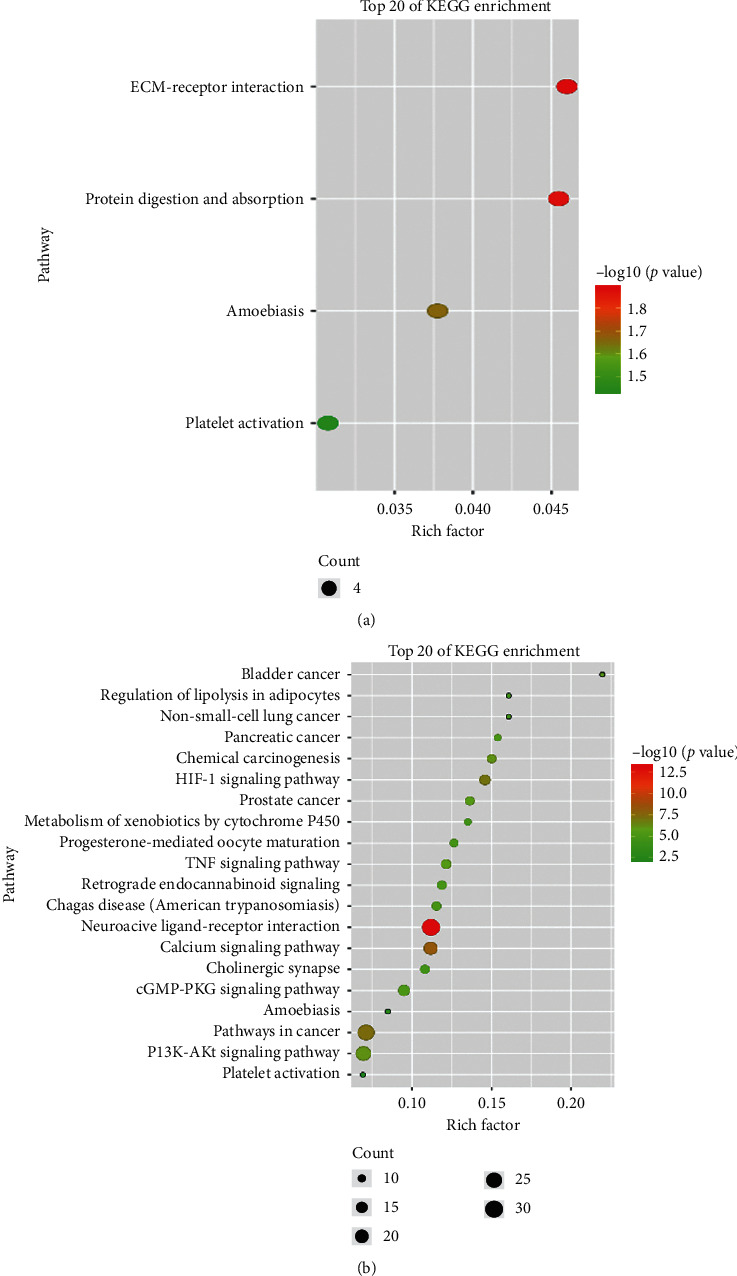
(a) KEGG signaling pathway analysis of DEGs of chronic gastritis patients peripheral blood leukocytes. (b) KEGG signaling pathway analysis of Sijunzi decoction potential targets.

To investigate the potential effect of Sijunzi decoction on peripheral blood leukocytes of chronic gastritis patients, we conducted GO and KEGG analyses of Sijunzi decoction and DEGs of chronic gastritis patients, respectively. The GO analysis showed that overlapped biological processes were angiogenesis, collagen catabolic process, extracellular matrix organization, collagen fibril organization, and tissue homeostasis (Figures 4(a) and 4(b)). The KEGG analysis showed that platelet activation and amoebiasis were the overlapped pathways (Figures 5(a) and 5(b)). These results suggest that Sijunzi decoction can also suppress the inflammation of peripheral blood leukocytes.

## 4. Discussion

Chronic gastritis is characterized with inflammatory response in the gastric mucosa [11]. The inflammatory cells are accumulated in the lamina propria [12]. The histological examination indicates that the inflammatory cells such as neutrophils and macrophages are infiltrated in the gastric epithelium, while the inflammatory cells are infiltrated in lamina propria [13]. *Helicobacter pylori* is one of the most important pathological factors leading to the chronic gastritis [14]. *Helicobacter pylori* could induce chronic immune response to form germinal centers. In the long term, gastric mucosa might transfer to small intestinal type mucosa. In the site of chronic gastritis, inflammatory cells and inflammatory mediators, including oxygen free radicals, form a vicious circle. Furthermore, stomach adenocarcinoma would occur in the site of metaplastic gastric mucosa [15]. So, it is important to alleviate inflammation in chronic gastritis at the early stage before its malignant transformation.

In the network pharmacology analysis of Sijunzi decoction and chronic gastritis, we identified 18 potential targets of Sijunzi decoction on chronic gastritis which can be categorized into immunologically related proteins and cancer-related proteins. In the immune-related targets, IL1B, IL2, TNF, and CCL2 were well-known proinflammatory cytokines. These inflammatory mediators could recruit and activate inflammatory cells, such as neutrophils and macrophages, to amplify inflammation [16]. MPO and SOD1 were involved in the oxidative stress [17]. PTGS1 and PTGS2 were related with prostaglandin, which is an important hormone in immune regulation [18]. The GO analysis of the overlap targets showed that the most related biological processes were positive regulation of nitric oxide biosynthetic process, positive regulation of ERK1 and ERK2 cascade, cellular response to drug, and negative regulation of apoptotic process and MAPK cascade. ERK cascade and MAPK cascade were related with inflammatory response in chronic gastritis. Nitric oxide biosynthetic process was related with oxidative stress [19]. Apoptosis plays important roles in the resolution of inflammation. Thus, these results indicated that Sijunzi decoction could ameliorate chronic gastritis by alleviating inflammation and oxidative stress. In addition, some overlap targets were considered as cancer-related genes. So, we inferred that Sijunzi decoction could also reduce the incidence rates of malignant transformation in chronic gastritis patients by inhibiting inflammatory response.

Then, we used the RNA-seq data of peripheral leukocytes from chronic superficial gastritis patients to investigate whether Sijunzi decoction can suppress the inflammation in the peripheral blood. After performing a functional analysis on 102 DEGs and Sijunzi decoction potential targets, we found that platelet activation, angiogenesis, and pathways in cancer were the potential target pathways of Sijunzi decoction on peripheral leukocytes of chronic superficial gastritis patients. Platelets participate in the inflammatory reactions and play important roles in the progression of inflammatory diseases. It has been shown that platelets express Toll-like receptors [20] and complement receptors [21]. Studies have shown that complement proteins can deposit on the surface of platelets, which are involved in immune function [21]. The formation of extracellular neutrophil traps also requires the help of Toll-like receptor 4 signaling in platelets [22]. In addition, platelets also synthesize inflammatory factors, such as interleukin-1*β* [23]. Platelet activating factor induced by *Helicobacter pylori* is one important cause of gastritis [24]. Besides, it has been proposed that there is a negative feedback mechanism that can inhibit the platelet reaction, which is mediated by the platelet-released NO after the activation [25].

Activated platelets can promote tumor cell growth, invasion, and angiogenesis [26]. Elevated platelets can be seen in various cancer types with poor prognosis, including colorectal cancer, gastric cancer, and ovarian cancer [27–29]. Angiogenesis is the biological process by which new blood vessels are formed. The activation of platelets can lead to angiogenesis. The ERK signaling and PI3K-Akt signaling can be activated by platelet-lysate in endothelial cell [30]. Activation of these pathways can cause the release of matrix metalloproteinases that are necessary for angiogenesis [31]. PI3K-Akt signaling pathway induces telomerase reverse transcriptase expression in chronic gastritis. The telomerase could be activated in the process of carcinogenesis [32]. Previous studies found that PI3K-Akt pathway was enhanced in gastric mucosa of stomach cancer, which was correlated with poor survival prognosis [33]. So, we speculated that Sijunzi decoction could alleviate chronic gastritis by inhibiting angiogenesis and inflammation, which might also have resulted in the reduction of the stomach cancer incidence.

Taken together, Sijunzi decoction alleviates chronic gastritis by regulating platelet activation, suppressing the local gastric inflammation and inflammations in peripheral blood leukocytes, and might also reduce the incidence of stomach cancer in chronic gastritis patients.

## 5. Conclusion

In this study, we explored the mechanism of Sijunzi decoction in the treatment of chronic gastritis. Firstly, we used online datasets to investigate the molecular targets and related pathways of Sijunzi decoction against chronic gastritis. Sijunzi decoction alleviated chronic gastritis by inhibiting inflammation and oxidative stress. Sijunzi decoction might also reduce the incidence of stomach cancer in chronic gastritis by suppressing inflammatory response. Then, we used RNA-seq data of peripheral blood leukocytes from chronic superficial gastritis patients to explore the impact of Sijunzi decoction on peripheral blood leukocytes. Our results showed that Sijunzi decoction can also suppress the inflammation of peripheral blood leukocytes via platelet activation and amoebiasis pathways. The present study could provide new insight into mechanisms of Sijunzi decoction in treating chronic gastritis.

## Figures and Tables

**Table 1:** The information of overlap targets about Sijunzi decoction and chronic gastritis.

Biological function	Target names
Immune-related function	IL1B, IL2, TNF, MPO, CCL2, SOD1, PTGS1, and PTGS2
Cancer-related function	PTPN11, TP53, BCL2, ODC1, GSTM1, PRKCH, EGFR, and VEGFA
Else	AHR, A2ML1

**Table 2:** Differentially expressed genes in peripheral blood leukocytes of chronic gastritis patients.

Expression	Genes
Upregulated genes	ADAMTSL5, AKAP3, AKR1C1, AQP1, BEGAIN, C10orf91, C14orf132, C4BPA, C4BPB, CNTD2, COL10A1, COL26A1, COL27A1, COL2A1, CPLX2, CRB3, CRMP1, CTGF, DPYSL3, EFNB2, HSD17B8, LILRB3, LOC102724436, LOC105373386, LOC105376526, MEF2B, MLF1, MSH5, MYL4, NAPRT, NEFL, OR1J2, PCDHGA11, PCDHGA8, PCDHGC5, PCSK1N, PHLDB1, PLEKHN1, PLGLB2, PPP1R1B, PSMB3, RXRB, SAMD14, SCN5A, SHISA8, SLC25A2, SLC25A29, SLMO1, SOGA3, SORCS3, SPP1, SYNGAP1, TMEM160, TMEM238, ZFP57, and ZNF683.
Downregulated genes	CCDC155, CFAP46, CLDN7, CLEC4C, COL5A3, CPXM1, EGFL8, EIF3K, FAM171B, HLA-E, HOXA3, HTR2B, IFNGR2, IL23R, INTS3, KCNK17, LOC101930332, LOC105375057, MATN2, NR1I2, NXN, PLCB4, POMZP3, PTPRB, PTPRR, PTPRT, PYGO1, RAMP1, RANBP17, SLC47A1, SLC4A10, SNX32, SPTLC3, TAS2R20, TAS2R46, TCTEX1D1, TMEM132B, TMEM171, TREML4, ZNF415, and ZP3.

## Data Availability

The data that support the findings of this study are available from the corresponding author upon request.
